# The correlation between ANAs and pregnancy loss and their impact on IVF/ICSI‐ET pregnancy outcomes in patients with recurrent pregnancy loss

**DOI:** 10.1002/ijgo.16183

**Published:** 2025-01-30

**Authors:** Manman Liu, Hebo Zhang, Shilian Xu, Rui Zhang, Mengfan Yuan, Bingnan Ren, Wenjuan Zhang, Zhaozhao Liu, Yichun Guan

**Affiliations:** ^1^ Department of Reproductive Medicine Third Affiliated Hospital of Zhengzhou University Zhengzhou China

**Keywords:** antinuclear antibody, assisted reproductive technology, pregnancy loss, recurrent pregnancy loss

## Abstract

**Objective:**

To explore the correlation between antinuclear antibodies (ANAs) and pregnancy loss (PL), and to observe its impact on the pregnancy outcomes of in vitro fertilization/intracytoplasmic sperm injection‐embryo transfer (IVF/ICSI‐ET) in recurrent PL (RPL) patients.

**Methods:**

In this retrospective study, patients first seen at the hospital between January 2016 and December 2022 and who underwent two ANA tests within 4–6 weeks were included. After exclusion of confounding factors, patients were allocated to the non‐pregnancy loss (non‐PL), single‐PL, or RPL group according to previous number of PLs, and the correlation between PL and ANAs was analyzed. The first embryo transfer (ET) after in vitro IVF/ICSI without immunological treatment was placed in the RPL group, and patients were classified into the ANA‐negative or ANA‐positive subgroup according to ANA titer. The effect of ANAs on pregnancy outcomes in the RPL patients after IVF/ICSI‐ET was further analyze.

**Results:**

The results of multivariate unordered logistic regression showed that when the non‐PL group was used as the reference, ANA positivity was an independent risk factor for RPL (*P* = 0.023) but not for single PL (*P* = 0.654). When the single‐PL group was used as the reference, ANA positivity was an independent risk factor for RPL (*P* = 0.022). Multivariate logistic regression analysis revealed that the early PL rate of the ANA‐positive subgroup was significantly higher than that of the ANA‐negative subgroup (*P* = 0.009), and the total PL rate of the ANA‐positive subgroup was significantly higher than that of the ANA‐negative subgroup (*P* = 0.049).

**Conclusion:**

The results showed that ANA positivity may be related to RPL occurrence, but there was no significant correlation between ANA positivity and single PL. ANA positivity is associated with PL occurrence in RPL patients after transfer, and the correlation is reflected mainly in the first trimester.

## Synopsis

The results of this study indicate that ANAs are associated with RPL and also with miscarriage in RPL patients after transfer.

## 1 INTRODUCTION

Pregnancy loss (PL) is a common complication of pregnancy in women of childbearing age, and its incidence rate is approximately 10%.^1^ According to the classical view, embryonic chromosome abnormalities are the main cause of PL, and the etiology of PL can account for 50% of cases.^2^ According to the guidelines of the European Society for Human Reproduction and Embryology (ESHRE) and the Royal College of Obstetricians and Gynecologists (RCOG),^3^, ^4^ the spontaneous demise of a pregnancy before 24 weeks of pregnancy is referred to as a PL, while in China, PL before 28 weeks of pregnancy is still defined as spontaneous abortion (SA).^5^ According to the ESHRE guidelines, recurrent PL (RPL) is defined as the loss of two or more pregnancies, including non‐visualized PL. In 2019, Green et al. reported that the incidence of RPL was approximately 1%–5%.^6^

In recent years, an increasing number of scholars have begun to pay attention to the role of immune factors in adverse pregnancy and childbirth outcomes, especially RPL.^7^, ^8^ For example, classical antiphospholipid syndrome (APS) is closely related to RPL,^9^, ^10^ and it is the only immunological disease recommended for examination and treatment.^3^, ^11^ Because the components and functions of the immune system vary and the interactions among various immune components are complex, people have gradually begun to study specific pathogenic autoantibodies,^12^ such as antiphospholipid antibodies, antinuclear antibodies (ANAs), and antithyroid autoantibodies.

Antinuclear antibodies are autoantibodies that are produced by the body, and they combine with nuclear and intranuclear antigens. Approximately 13.0% of healthy people are positive for ANAs, but the clinical significance of this phenomenon is not clear at present.^13^ ANAs are also present in several immunological diseases, such as systemic lupus erythematosus (SLE) and Sjögren's syndrome (SS), and these diseases can also lead to adverse pregnancy outcomes.^14^, ^15^ However, the role of simple ANA positivity in single PL and RPL is unclear.

A study by Zhu in 2013 showed that ANA positivity may affect the outcomes of in vitro fertilization/intracytoplasmic sperm injection‐embryo transfer (IVF/ICSI‐ET), but this study did not limit the number of PLs in patients.^16^ A retrospective study by Sakthiswary showed that the percentage of ANA‐positive patients with unexplained RPL (URPL) was significantly higher than that of healthy controls.^17^ Recent meta‐analyses have shown that the rate of ANA positivity in RPL patients is higher than that in non‐RPL patients^18^, ^19^; however, these meta‐analyses did not define the number of PLs in non‐RPL patients, and the control population included in each study differed. Therefore, we conducted this retrospective cohort study to explore the association between ANAs and PL and to evaluate its effect on IVF/ICSI‐ET pregnancy outcomes in RPL patients.

## 2 MATERIALS AND METHODS

### 2.1 Study design and subjects

This retrospective observational case–control study included patients who visited the Department of Reproductive Medicine at the Third Affiliated Hospital of Zhengzhou University for the first time from January 2016 to December 2022 and were tested for ANAs twice within 4–6 weeks. The number of PLs was documented as the total number before the first ANA test. To reduce the influence of confounding factors, patients were excluded if they had any of the following: (1) chromosome karyotype abnormality in either spouse; (2) abnormal anatomical structure of the uterus, such as bicornuate uterus, saddle uterus, septate uterus, uterine fibroids, or adenomyosis; (3) endometrial lesions and hydrosalpinx; (4) abnormal endocrinology or metabolism; (5) immunological diseases, such as APS, SLE, or SS; (6) prethrombotic state; (7) recurrent implantation failure (RIF); or (8) different ANA test results. These patients were divided into the non‐PL group (no previous PL), single‐PL group (history of one previous PL), and RPL group according to the number of previous PLs to evaluate whether ANAs are a risk factor for PL.

Due to the fact that some ANA‐positive patients received hydroxychloroquine treatment after consultation with rheumatologists, this study selected untreated patients in the RPL group who received their first transplant after undergoing IVF/ICSI. According to the ANA test results, the patients were divided into the ANA‐positive subgroup and the ANA‐negative subgroup to analyze the impact of ANA on pregnancy outcomes after transfer. In addition, to reduce confounding factors, the study excluded patients who used donor sperm or eggs or who underwent IVF/ICSI with preimplantation genetic testing.

In this paper, PL is defined as natural fetal death before 24 weeks of pregnancy, and RPL is defined as PL occurring two or more times, including non‐visualized PL, according to the ESHRE guidelines.^3^ RIF is defined as failure to achieve pregnancy after three consecutive high‐quality embryos.^20^

### 2.2 ANA detection method

Considering the experimental error, ANA detection should be carried out at least twice, with an interval of 4–6 weeks. It is clinically significant that the results of the two tests are the same.

#### 2.2.1 Detection of ANAs based on indirect immunofluorescence

Fresh blood samples should be used. Blood samples containing particulate matter were centrifuged at low speed (<1000× *g*) to remove particles and then used within the first 8 h after serum separation. In the first incubation, the serum was diluted to 1:32, 1:100 or higher, and the combination of HEp‐2 cells and frozen sections of animal tissues was used as the matrix. Then, 35 μL of diluted serum was added and incubated at 20–26°C for 30 min. For the second incubation, the cells were gently washed with distilled water diluted with 1:10 cleaning buffer and then soaked in a dyeing dish three times for 5 min each. Then, 35 μL of fluorescein isothiocyanate‐conjugated secondary antibody was added to the sample, and the sample was incubated at 20–26°C for 30 min, washed (as described earlier), and sealed on the tablet. The results were immediately obtained by fluorescence microscopy. Each test and result interpretation was performed with reference to positive and negative controls. When weak fluorescence appeared in the nucleus (titers between 1:80 and negative) or if the experimental results were uncertain, the samples were evaluated again.

### 2.3 Observation indicators and their definitions

The clinical pregnancy rate was defined as the number of clinically pregnant patients/the number of total transplantation patients × 100%. The presence of one or more gestational sacs on ultrasound indicated a clinical pregnancy, including an intrauterine pregnancy, an ectopic pregnancy, a simultaneous intrauterine and extrauterine pregnancy, or pregnancy with only a gestational sac but no fetal heartbeat.

The early PL rate was defined as the number of PL patients within 12 weeks of pregnancy/the number of clinically pregnant patients × 100%.

The late PL rate was defined as the number of PL patients after 12 weeks and before 24 weeks of pregnancy/the number of clinically pregnant patients × 100%.

The total PL rate was defined as the number of PL patients before 24 weeks of pregnancy/the number of clinically pregnant patients × 100%.

The ectopic pregnancy rate was defined as the number of ectopic pregnancy patients/the number of clinical pregnancy patients × 100%. An ectopic pregnancy was defined as a pregnancy in which a fertilized egg was implanted outside the uterine cavity, including tubal pregnancy, ovarian pregnancy, cervical pregnancy, broad ligament pregnancy, and abdominal pregnancy.

The premature birth rate was defined as the number of patients with live births between 24 and 37 weeks of pregnancy/the number of patients with live births after 24 weeks of pregnancy.

The live birth rate was defined as the number of patients with live births after 24 weeks of pregnancy/total number of transplantation patients × 100%.

### 2.4 Statistical analysis

SPSS 26.0 software was used for statistical analysis. Normally distributed data with homogeneous variance are expressed as the mean ± standard deviation ($\bar{x}\pm s$). Student's *t*‐test was used for comparisons between two groups, and one‐way analysis of variance was used for comparisons among multiple groups. Non‐normal distribution and/or homogeneity of variance are expressed as medians (quartiles) [M (Q1, Q3)]. The Kruskal–Wallis rank‐sum test was used for comparisons between groups, with a significance level of *α* = 0.05. Count data are expressed as the rate or composition ratio (%). The chi‐square test was used for comparisons between groups. When the theoretical frequency was less than 5, the Fisher exact probability method was used for comparisons between groups, and the significance level was set at *α* = 0.05. The Bonferroni method was used for pairwise comparisons between multiple groups of measurement and counting data, and the corrected test level was *α*′ = 0.0167. Variance inflation factor (VIF) selection was used to test collinearity between variables, and the VIF of all variables was less than 5, suggesting that there was no obvious multicollinearity. Factors related to the PL were analyzed by multivariate unordered logistic regression, and variables with *P* < 0.05 among multiple groups were included. Univariate and multivariate logistic regression analyses were used to correct for confounding factors related to the clinical pregnancy outcomes of RPL patients. When the clinical characteristics from the univariate logistic regression analysis showed *P* < 0.10, they were included in the multivariate logistic regression analysis.

### 2.5 Ethical approval

All the procedures of this study conformed to the 1964 Helsinki Declaration and its later amendments or similar ethical standards and passed the examination and approval of the Ethics Committee of the Third Affiliated Hospital of Zhengzhou University (ethics no.: 2023‐067‐01). The need to obtain informed consent was waived by the Ethics Committee of the Third Affiliated Hospital of Zhengzhou University.

## 3 RESULTS

### 3.1 Characteristics of all the subjects

**FIGURE 1:**
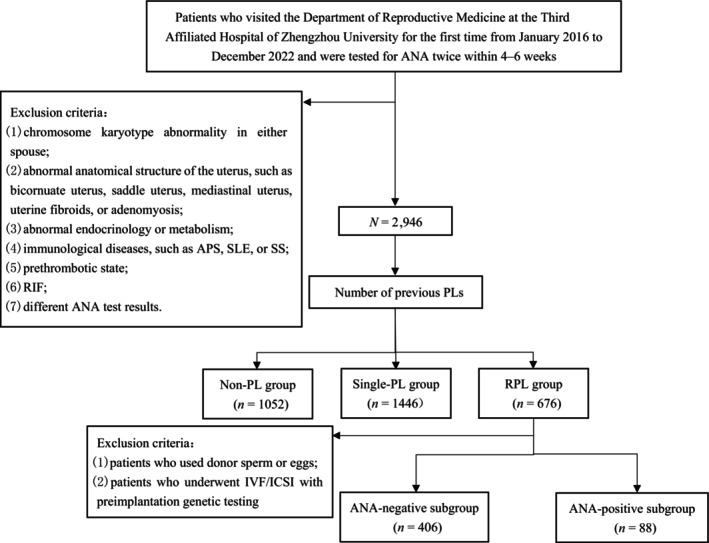
Research flowchart. ANA, antinuclear antibody; APS, antiphospholipid syndrome; IVF/ICSI, in vitro fertilization/intracytoplasmic sperm injection‐embryo transfer; PL, pregnancy loss; RIF, recurrent implantation failure; SLE, systemic lupus erythematosus; SS, Sjögren's syndrome.

A total of 3174 patients, including 1052 patients in the non‐PL group (33.14%), 1446 patients in the single‐PL group (45.56%), and 676 patients in the RPL group (21.30%), were enrolled in this study (Figure 1). Among the three groups, there were significant differences in male age, infertility duration, and infertility factors (all *P* < 0.05). There was no statistically significant difference in other clinical features among the three groups (*P* > 0.05). There were 157 ANA‐positive patients in non‐PL group, 244 patients in the single PL group, and 139 patients in the RPL group. The difference in the percentage of ANA‐positive individuals among the three groups was significant (14.92% vs. 16.87% vs. 20.65%, *P* = 0.010), with the RPL group having a significantly higher ANA‐positive rate than the non‐PL group (*P* < 0.0167) (Table 1). There was no significant difference in the proportion of patients according to ANA titer among the three groups (*P* = 0.897) (Table 2).

**TABLE 1:** Comparisons of the characteristics of the included patients.

Characteristics	Non‐PL group	Single‐PL group	RPL group	*P‐*value
*N*	1052	1446	676	
Female age (year)	33.30 ± 4.96	33.15 ± 4.88	33.34 ± 5.06	0.652
Male age (year)	33.00 (30.75–37.00)	34.00 (31.00–38.00)^a^	35.00 (32.00–40.00)^b^	<0.001
Female BMI	23.69 ± 3.17	23.90 ± 3.22	23.79 ± 3.26	0.542
Infertility duration	4.00 (2.00–6.00)	1.00 (0.60–2.50)^a^	1.00 (0.80–2.50)^a^	<0.001
bFSH	6.62 (5.49–8.04)	6.70 (5.49–8.19)	6.82 (5.79–8.37)	0.070
AMH	18.41 (9.08–32.40)	18.92 (8.27–31.83)	17.91 (7.20–32.99)	0.160
AFC	16.00 (9.75–23.25)	16.00 (10.00–23.00)	16.00 (10.00–24.00)	0.072
Infertility factor				<0.001
Female factor	358 (34.03%)	670 (46.33%)^a^	350 (51.78%)^a^	
Male factor	104 (9.89%)	187 (12.93%)	66 (9.76%)	
Factor of both sides	117 (11.12%)	274 (18.95%)^a^	76 (11.24%)^b^	
Unknown cause	473 (44.96%)	315 (21.78%)^a^	184 (27.22%)^a^, ^b^	
ANA status				0.010
ANA‐positive	157 (14.92%)	244 (16.87%)	139 (20.56%)^a^	
ANA‐negative	895 (85.08%)	1202 (83.13%)	537 (79.44%)^a^	

Abbreviations: AFC, antral follicle count; AMA, anti‐Mullerian hormone; ANA, antinuclear antibodies; bFSH, basic follicle stimulating hormone; BMI, body mass index (calculated as weight in kilograms divided by the square of height in meters); PL, pregnancy loss; RPL, recurrent pregnancy loss.

^a^ *P* < 0.0167 compared with the non‐PL group.

^b^ *P* < 0.0167 compared with the single‐PL group.

**TABLE 2:** The proportions of patients according to antinuclear antibody (ANA) titer in each group of ANA‐positive patients.

	Non‐PL group	Single‐PL group	RPL group	*P*‐value
*n*	157	244	139	
ANA titer				0.897
1:80	118 (75.16%)	180 (73.77%)	100 (71.94%)	
1:160	23 (14.65%)	43 (17.62%)	25 (17.99%)	
≥1:320	16 (10.19%)	21 (8.61%)	14 (10.07%)	

Abbreviations: PL, pregnancy loss; RPL, recurrent pregnancy loss.

### 3.2 Multivariate unordered logistic regression analysis of pregnancy loss

Multivariate unordered logistic regression was performed in the study, with the non‐PL group and single‐PL group as controls. When the non‐PL group was used as the reference, ANA positivity was an independent risk factor for patients in the RPL group (adjusted odds ratio [aOR] 1.427, 95% confidence interval [CI] 1.050–1.940, *P* = 0.023) but not for patients in the single‐PL group (aOR 1.063, 95% CI 0.814–1.387, *P* = 0.654) (Table 3). Similarly, when the single‐PL group was used as the reference, ANA positivity was an independent risk factor for patients in the RPL group (aOR 1.343, 95% CI 1.043–1.729; *P* = 0.022) (Table 4).

**TABLE 3:** The results of the multivariate unordered logistic regression analysis with the non‐pregnancy loss (non‐PL) group as the control.

Characteristics	Single‐PL group			RPL group		
	*β*‐value	Adjusted OR (95% CI)	*P*‐value	*β*‐value	Adjusted OR (95% CI)	*P*‐value
Female age	0.040	1.041 (1.007–1.077)	0.018	0.059	1.061 (1.020–1.105)	0.004
Male age	0.038	1.038 (1.008–1.070)	0.013	0.045	1.046 (1.011–1.083)	0.010
Female BMI	0.025	1.025 (0.995–1.056)	0.103	0.117	1.124 (1.085–1.165)	<0.001
Infertility duration	−0.352	0.703 (0.676–0.732)	<0.001	−0.435	0.647 (0.611–0.686)	<0.001
AMH	0.002	1.002 (0.998–1.007)	0.328	0.005	1.005 (0.999–1.010)	0.107
AFC	−0.010	0.990 (0.975–1.006)	0.213	−0.029	0.972 (0.956–0.988)	0.001
Infertility factors						
Female factor	Ref.			Ref.		
Male factor	0.101	1.106 (0.812–1.506)	0.523	−0.189	0.828 (0.558–1.229)	0.349
Factor of both sides	0.513	1.670 (1.235–2.258)	0.001	−0.193	0.824 (0.560–1.213)	0.327
Unknown cause	−0.872	0.418 (0.337–0.519)	<0.001	−0.726	0.484 (0.372–0.629)	<0.001
ANA status						
ANA‐negative	Ref.			Ref.		
ANA‐positive	0.061	1.063 (0.814–1.387)	0.654	0.356	1.427 (1.050–1.940)	0.023

Abbreviations: AFC, antral follicle count; AMH, anti‐Mullerian hormone; ANA, antinuclear antibodies; BMI, body mass index (calculated as weight in kilograms divided by the square of height in meters); CI, confidence interval; OR, odds ratio; PL, pregnancy loss; RPL, recurrent pregnancy loss.

**TABLE 4:** The results of multivariate unordered logistic regression with the single‐pregnancy loss (PL) group as the control group.

Characteristics	RPL group		
	*β*‐value	Adjusted OR (95% CI)	*P*‐value
Female age	0.019	1.019 (0.986–1.054)	0.263
Male age	0.008	1.008 (0.980–1.036)	0.582
Female BMI	0.092	1.097 (1.064–1.131)	<0.001
Infertility duration	−0.083	0.920 (0.869–0.975)	0.005
AMH	0.002	1.002 (0.998–1.007)	0.330
AFC	−0.027	0.973 (0.985–0.989)	0.001
Infertility factors			
Female factor	Ref.		
Male factor	−0.289	0.749 (0.537–1.043)	0.087
Factor on both sides	−0.706	0.493 (0.363–0.670)	<0.001
Unknown cause	0.146	1.157 (0.912–1.469)	0.229
ANA status			
ANA‐negative	Ref.		
ANA‐positive	0.295	1.343 (1.043–1.729)	0.022

Abbreviations: AFC, antral follicle count; AMH, anti‐Mullerian hormone; ANA, antinuclear antibodies; BMI, body mass index (calculated as weight in kilograms divided by the square of height in meters); CI, confidence interval; OR, odds ratio; RPL, recurrent pregnancy loss.

### 3.3 Characteristics of RPL patients receiving IVF/ICSI‐ET

After the patients were screened according to the exclusion criteria in the RPL group, 406 were included in the ANA‐negative subgroup, and 88 were included in the ANA‐positive subgroup. There was no statistically significant difference in clinical characteristics between the two subgroups (all *P* > 0.05) (Table 5).

**TABLE 5:** Comparison of the characteristics of recurrent pregnancy loss (RPL) patients receiving in vitro fertilization/intracytoplasmic sperm injection‐embryo transfer (IVF/ICSI‐ET).

Characteristics	ANA‐negative subgroup	ANA‐positive subgroup	*P*‐value
*n*	406	88	
Female age (year)	34.20 ± 4.30	33.57 ± 3.29	0.191
Male age (year)	35.00 (32.00–39.00)	37.00 (32.00–40.00)	0.094
Female BMI	23.78 (21.78–25.89)	24.25 (22.23–25.20)	0.977
Number of pregnancy losses	2.00 (2.00–2.00)	2.00 (2.00–3.00)	0.679
Infertility duration	1.00 (0.60–2.00)	1.00 (0.60–2.00)	0.415
bFSH	6.70 (5.52–8.05)	6.96 (5.81–8.30)	0.229
AMH	16.48 (8.18–32.94)	12.11 (8.25–25.11)	0.155
AFC	15.00 (9.00–23.00)	14.00 (9.00–19.00)	0.077
Endometrial thickness on the day of transplantation	8.99 ± 1.84	9.04 ± 1.68	0.773
Proportion of single‐embryo transfers	50.49 (205/406)	54.55 (48/88)	0.490
Infertility factors			0.754
Female factor	192 (47.29%)	40 (45.45%)	
Male factor	214 (52.71%)	48 (54.55%)	
Factor on both sides			0.133
Unknown cause	205 (50.49%)	53 (60.23%)	
Type of embryo transfer	41 (10.10%)	12 (13.64%)	
Cleavage‐stage embryo transfer	54 (13.30%)	8 (9.09%)	
Blastocyst embryo transfer	106 (26.11%)	15 (17.05%)	
Embryo transfer cycle			0.530
Fresh cycle	89 (21.92%)	22 (25.00%)	
Thawing cycle	317 (78.08%)	66 (75.00%)	

Abbreviations: AFC, antral follicle count; AMH, anti‐Mullerian hormone; ANA, antinuclear antibodies; bFSH, basic follicle stimulating hormone; BMI, body mass index (calculated as weight in kilograms divided by the square of height in meters).

### 3.4 Pregnancy outcomes of RPL patients receiving IVF/ICSI‐ET

Because of the significant differences in the characteristics of the two subgroups, univariate and multivariate logistic regression were used to correct for confounding factors. Multivariate logistic regression analysis revealed that the early PL rate of patients in the ANA‐positive subgroup was significantly higher than that in the ANA‐negative subgroup (27.27% vs. 14.64% [aOR 3.012, 95% CI 1.310–6.921, *P* = 0.009]). There was no significant difference in the late PL rate between the two subgroups (4.55% vs. 5.02% [aOR 1.083, 95% CI 0.227–5.165, *P* = 0.920]), but the total PL rate of the ANA‐positive subgroup was still significantly higher than that of the ANA‐negative subgroup (31.82% vs. 19.67% [aOR 2.242, 95% CI 1.003–5.011, *P* = 0.049]). Univariate regression analysis revealed that the live birth rate of the ANA‐positive subgroup was significantly lower than that of the ANA‐negative subgroup (34.09% vs. 46.06% [OR 0.606, 95% CI 0.374–0.981, *P* = 0.042]). However, after multivariate logistic regression correction for confounding factors, there was no significant difference in the live birth rate between the two subgroups (aOR 0.652, 95% CI 0.368–1.103, *P* = 0.111) (Table 6).

**TABLE 6:** Univariate and multivariate logistic regression analyses of pregnancy outcomes in recurrent pregnancy loss (RPL) patients receiving in vitro fertilization/intracytoplasmic sperm injection‐embryo transfer (IVF/ICSI‐ET).

	ANA‐negative subgroup	ANA‐positive subgroup	Unadjusted OR (95% CI)	*P*‐value	Adjusted OR (95% CI)	*P*‐value
Clinical pregnancy rate	239/406 (58.87%)	44/88 (50.00%)	0.699 (0.440–1.109)	0.129	0.839 (0.508–1.386)	0.949
Early pregnancy loss rate^a^	35/239 (14.64%)	12/44 (27.27%)	2.168 (1.028–4.646)	0.042	3.012 (1.310–6.921)	0.009
Late pregnancy loss rate^a^	12/239 (5.02%)	2/44 (4.55%)	0.901 (0.195–4.171)	0.894	1.083 (0.227–5.165)	0.920
Total pregnancy loss rate^a^	47/239 (19.67%)	14/44 (31.82%)	1.906 (0.937–3.878)	0.075	2.242 (1.003–5.011)	0.049
Live birth rate	187 /406 (46.06%)	30/88 (34.09%)	0.606 (0.374–0.981)	0.042	0.652 (0.386–1.103)	0.111

Abbreviations: AFC, antral follicle count; AMH, anti‐Mullerian hormone; ANA, antinuclear antibodies; bFSH, basic follicle stimulating hormone; BMI, body mass index (calculated as weight in kilograms divided by the square of height in meters); CI, confidence interval; OR, odds ratio; RPL, recurrent pregnancy loss.

^a^ Represents the denominator of clinical pregnancy patients. Clinical pregnancy rate: male age, female BMI, bFSH, AMH, endometrial thickness on the day of transplantation, and proportion of single embryo transfer were covariates. Early pregnancy loss rate: female age, female BMI, and proportion of single embryo transfer were covariates. Late pregnancy loss rate: female BMI and proportion of single embryo transfer were covariates. Total pregnancy loss rate: female age, BMI, bFSH, and proportion of single embryo transfer were covariates. Live birth rate: female age, female BMI, bFSH, AMH, and AFC were covariates.

## 4 DISCUSSION

During mitosis, some substances in the nucleus may be exposed to the cell surface. When the body's immune system is out of balance, these substances may activate the autoimmune system and lead to the production of ANAs.^21^ In 1972, Abrahams et al. first described the relationship between ANAs and RPL.^22^ Recent studies have shown that ANAs may be related to adverse pregnancy outcomes, such as infertility and RPL^23^, ^24^; however, this conclusion remains controversial, and the relationship between ANAs and single PL is still unclear.

The results of multivariate unordered logistic regression in this study showed that ANAs were significantly correlated with RPL but not significantly correlated with single PL (Tables 3 and 4). However, the specific mechanism by which ANAs lead to RPL is still unclear. Previous studies have shown that ANAs in follicular fluid can negatively affect IVF/ICSI transplantation outcomes by decreasing oocyte quality or the number of invading granulosa cells.^25^, ^26^ However, the distribution of ANAs in serum and follicular fluid is inconsistent. In the study by Ying et al., ANAs were detected in the serum in 50 patients, but in the follicular fluid of only 36 patients.^27^ Veglia et al. injected ANA immunoglobulin G (IgG) into mice, which activated the complement system and resulted in PL.^28^ In addition, the precipitation of immune complexes at the maternal‐fetal interface may be one of the mechanisms leading to PL in ANA‐positive women.^29^, ^30^

Antinuclear antibody test results are usually displayed according to titer. The ANA titers of most of the ANA‐positive patients in the three groups of this study were 1:80, and there was no significant difference in the titer ratio (Table 2). Previous studies by Zhu et al. showed that there was no significant difference in IVF/ICSI outcomes between patients with an ANA titer >1:320 and patients with an ANA titer <1:320,^16^ indicating that the effect of ANAs on PL may be related only to whether or not the patient is positive for ANAs, and an increase in the ANA titer may not increase the PL rate.

After receiving IVF/ICSI‐ET, the early PL rate and total PL rate in the ANA‐positive subgroup of RPL patients were significantly higher than those in the ANA‐negative subgroup (Table 6). This indicates a correlation between ANA positivity and the occurrence of PL in RPL patients after transfer, which can be mutually confirmed with previous research results and further suggests that ANA positivity may be related to the occurrence of RPL. Notably, there was no significant difference in the late PL rate between the two subgroups, indicating that the correlation between ANAs and PL is reflected mainly in the first trimester of pregnancy (before 12 weeks). This result indirectly proves that after successful progression through the early stage of pregnancy, if the body does not undergo drastic immunological changes, PL will not occur due to immunological factors. Univariate logistic regression analysis of the live birth rate revealed that the live birth rate of the ANA‐positive subgroup was significantly lower than that of the ANA‐negative subgroup. After adjustments were made for the confounding factors of the live birth rate by multivariate logistic regression, the difference between the two groups become non‐significant (Table 6). This phenomenon may be due to the insufficient sample size in the study. A total of 88 patients were included in the ANA‐positive subgroup, and only 30 patients had live births. It is necessary to expand the sample size to further evaluate the live birth rate.

It is controversial whether patients with a previous history of PL need to be screened and treated for ANAs. According to the results of this study, we recommend screening and treatment for RPL patients. For patients with a history of only one PL, screening of ANAs should not be performed.

The advantage of this study lies in the addition of a single‐PL group, in which both the correlation between RPL and ANAs and the relationship between single PL and ANAs were investigated. The limitation of this study is that it was a retrospective study, and the occurrence of selection bias could not be avoided. Second, the etiology of embryo implantation failure is very similar to that of PL. In this study, only patients with RIF were excluded, and a history of occasional implantation failure, which can be used as a confounding factor to influence the research results, was not considered. In addition, due to insufficient sample size, this study did not conduct further subgroup analysis based on ANA titer. High‐quality research is needed to confirm the impact of ANA titer on the pregnancy outcomes of RPL patients.

## 5 CONCLUSION

The results showed that there was a correlation between ANAs and RPL, but there was no significant correlation between ANAs and single PL. ANA positivity was related to the occurrence of PL after RPL transplantation, and the correlation was reflected mainly in the first trimester of pregnancy.

## AUTHOR CONTRIBUTIONS

ML and HZ designed the study, analyzed the data, and drafted the manuscript, both of which have equal contribution. SX, RZ and MY participated in the critical discussion and revision of the article. BR, ZL, and YG assisted in the article writing and revision. The authors contributed to the article and approved the submitted version.

## FUNDING INFORMATION

This project has received funding from National Key Research and Development Program of China (2021YFC2700602) and the Henan Province Medical Science and Technology Research Program (Joint Construction) project (LHGJ20190369) in China.

## CONFLICT OF INTEREST STATEMENT

The authors have no conflicts of interest.

## Data Availability

The datasets generated and analyzed during the current study are not publicly available due to hospital's privacy policy but are available from the corresponding author on reasonable request.
